# Sibship, physical activity, and sedentary behavior: a longitudinal, observational study among Mexican-heritage sibling dyads

**DOI:** 10.1186/s12889-019-6521-y

**Published:** 2019-02-14

**Authors:** Kelly R. Ylitalo, Christina N. Bridges, Mariela Gutierrez, Joseph R. Sharkey, M. Renée Umstattd Meyer

**Affiliations:** 1 Robbins College of Health and Human Sciences, Baylor University, One Bear Place #97343, Waco, TX 76798-7343 USA; 2 Texas A&M School of Public Health, College Station, College Station, TX USA

**Keywords:** Physical activity, Sedentary behavior, Sibling dyads, Mexican-heritage siblings

## Abstract

**Background:**

Shared genetic and environmental factors suggest that family relationships are important predictors of obesity-related behaviors, yet little is known about how siblings influence physical activity and sedentary behaviors. This study examined physical activity and sedentary behavior between sibling dyads across summer and fall time points and determined if birth order and gender modify the relationship between sibling behaviors.

**Methods:**

Mexican-heritage families residing in *colonias* along the United States-Mexico border were recruited using *promotoras de salud* to participate in summer and school year surveys. Eighty-seven sibling dyads had complete data for the physical activity sub-study: 21 older brother-younger brother, 21 older brother-younger sister, 23 older sister-younger brother, and 22 older sister-younger sister dyads. Physical activity and sedentary behavior were measured using a validated 7-day recall instrument to create summary measures of weekly active, moderate-to-vigorous physical activity (MVPA) metabolic equivalents (MET), sitting, and screen time minutes. We used linear regression analyses to examine changes over time and the association between older and younger sibling behavior.

**Results:**

During summer, older siblings (mean age = 11.2 years) reported 1069 active minutes and 1244 sitting minutes per week; younger siblings (mean age = 8.3 years) reported 1201 active minutes and 1368 sitting minutes per week. Younger brothers reported fewer active minutes (mean = − 459.6; *p* = 0.01) and fewer MVPA MET-minutes (mean = − 2261.7; *p* = 0.02) of physical activity during the fall. Within all 87 dyads, older sibling physical activity was significantly associated with younger sibling active minutes (B = 0.45;*p* = 0.004) and MET-minutes (B = 0.45;*p* = 0.003) during summer but not fall; older sibling sedentary behavior was significantly associated with younger sibling sitting (B = 0.23;*p* = 0.01) and screen time minutes (B = 0.23;p = 0.004) during fall but not summer. After stratifying by gender dyad groups, younger brother behavior was strongly associated with older brother behavior at both time points.

**Conclusion:**

Younger siblings appear to emulate the physical activity behaviors of their older siblings during non-school summer months and sedentary behaviors of older siblings during school-time fall months, especially older brother-younger brother dyads. Family-based interventions to increase physical activity and decrease sedentary behavior are growing in popularity, but more work is needed to understand the role of sibling influences.

## Background

Siblings are our first friends in life. Almost 80% of adults in the United States have some kind of sibling [1], and these peer relationships often represent some of the longest-lasting and most influential relationships throughout the life course. Shared genetic, environmental, social, and behavioral factors within families, and sibships in particular, suggest that these relationships are important considerations for health and obesity-related research. For example, children with one overweight parent are more likely to be overweight compared to children of normal-weight parents [2], only children are more likely to be obese than those with siblings [3], and younger siblings of obese older siblings are five times more likely to be obese even after accounting for parental obesity [4].

A growing body of physical activity literature has focused on family-based measurement and intervention. For example, within parent-child dyads, reciprocal encouragement is positively correlated with co-participation in physical activity [5] and parenting strategies such as reinforcement and limit setting can promote physical activity and reduce sedentary time among adolescents [6]. However, little is known about physical activity and sedentary behaviors within sibships, who arguably share more social similarities than parent-child dyads.

Two competing theories may explain the relationship between sibling physical activity and sedentary behaviors. The *social cognitive theory* posits that younger siblings emulate the behaviors of the older siblings [7], which suggests a *positive* relationship between physical activity and sedentary behavior would be observed in comparisons of younger and older siblings. Conversely, the *de-identification theory* posits that younger siblings attempt to differentiate themselves from their older siblings [8], which suggests an *inverse* relationship between younger and older sibling behavior comparisons. Results on physical activity within adolescent sibships are mixed and limited [9, 10], and it is unclear how the size, gender, and age composition of the sibship may influence the strength and direction of physical activity and sedentary behavior concordance.

Mexican-heritage families represent an important opportunity to evaluate physical activity among sibships because they tend to report high levels of familism and siblings are encouraged to support and value one another [11]. However, Mexican-heritage families living along the Texas-Mexico border also have high rates of obesity [12] and engage in less physical activity and more sedentary behavior than their non-Hispanic white counterparts [13], which means they are a priority population for health promotion efforts. The primary purpose of this study was to compare physical activity and sedentary behavior between older and younger siblings within Mexican-heritage sibling dyads, across school year and non-school year time points, and determine if the gender composition of sibling dyads modified the relationship between older and younger sibling behaviors.

## Methods

### Design

Mexican-heritage families residing in *colonias* (unregulated settlements with informal housing) [14] in Hidalgo County, Texas along the United States-Mexico border were recruited by *promotoras de salud*. *Promotora-*researchers are trained and/or certified community health workers employed in Hispanic and Latina/o communities who received additional training in research methodologies [15]. For this study, eligible households had two sibling children between 5 and 14 years of age, one of which was between 7 and 11 years of age, both residing in the household; the mother of the siblings resided in the home; and both children and mothers spoke Spanish. Youth participants and their mothers were asked to enroll in a longitudinal, observational study and complete at-home surveys delivered once during the summer (June–July; non-school year) and once during the fall (August–December; during the school year) of a traditional school year. Surveys were interviewer-administered to mothers and children independently by *promotora*-researchers. All surveys were translated using a translation-back-translation method and administered in Spanish as was the preferred language within each home.

### Measures

Youth physical activity and sedentary behaviors were assessed using a validated 7-day recall instrument with pictures of physical and sedentary activities [16]. Youth were asked whether they had participated in each of the behaviors and for how many days and minutes they performed each activity. Original scale development indicates child and parent-proxy report were similar and demonstrated a significant correlation between the child and parent report (rho = 0.44, *p* < 0.05) [16]. The Compendium of Metabolic Equivalents for children [17] was used to categorize physical activity as light (1.6–2.9 metabolic equivalent units [METs]), moderate (3–6 METs), and vigorous activities (> 6.0 METs) [18]. Created summary measures for physical activity behaviors included weekly minutes of activity and weekly moderate-to-vigorous physical activity (MVPA) MET-minutes. Created summary measures for sedentary behaviors included weekly minutes of sitting time and weekly minutes of screen time, defined as sitting time spent using an electronic device such as television viewing, videogames, or texting.

Other sociodemographic measures included youth age, gender, and nativity (United States or Mexico). Mothers were asked questions about age, nativity, educational attainment (in total years), and marital status (not married, married, or living with a partner but not married).

### Statistical analysis

Descriptive statistics including frequencies, proportions, and means were calculated for all sociodemographic variables for both mothers and children. Winsorizing was utilized to correct outliers common with self-reported physical activity data by top-coding the top 10% with the 90th percentile, consistent with other investigations [19]. For example, before winsorizing, older children reported 1350 mean active minutes per week during summer; after winsorizing, active minutes per week were reduced to a mean of 1069 min, but median active minutes per week were not affected by winsorizing. We report the winsorized data in all results and tables. Physical activity, measured with active minutes per week and MET-minutes per week, and sedentary behavior, measured with sitting minutes per week and minutes of screen time per week, was reported for both summer and fall time points. We tested within-child changes over time between summer and fall time periods using Student’s t tests. We compared older sibling behavior and younger sibling behavior within all 87 dyads using linear regression, and then stratified the within-dyad comparisons by birth order-gender groups (older brother-younger brother, older brother-younger sister, older sister-younger brother, or older sister-younger sister). We report the adjusted R^2^ from regression models to describe the proportion of variance in younger sibling behavior that is explained by older sibling behavior. Data management and analyses were conducted with SAS v9.4 (SAS Institute Inc., Cary, NC) and statistical significance was two-sided at the α = 0.05 level.

## Results

In total, 174 children (87 sibling pairs or dyads) contributed complete data: 21 older brother-younger brother (24.1%), 21 older brother-younger sister (24.1%), 23 older sister-younger brother (26.4%), and 22 older sister-younger sister dyads (25.3%). Many older siblings (80.7%) and younger siblings (93.1%) were born in the United States (Table 1), while only 10.3% of mothers were born in the United States (Table 2). Over two-thirds of mothers completed 9 or fewer years of education, and approximately half were married.

**Table 1:** Demographic characteristics of children by sibling birth order (*n* = 174)

	Older Sibling (*n* = 87)		Younger Sibling (*n* = 87)	
	n (%)	years (std)	n (%)	years (std)
Age		11.2 (1.6)		8.3 (1.6)
Gender				
Male	42 (48.3)		44 (50.6)	
Female	45 (51.7)		43 (49.4)	
Nativity				
United States	70 (80.5)		81 (93.1)	
Mexico	17 (19.5)		6 (6.9)

**Table 2:** Demographic characteristics of mothers (*n* = 87)

	n (%)	years (std)
Age		35.5 (7.0)
Nativity		
United States	9 (10.3)	
Mexico	78 (89.7)	
Education		
< 7 years	28 (32.2)	
7 to 9 years	32 (36.8)	
10 to 11 years	13 (14.9)	
12 or more years	14 (16.1)	
Marital Status		
Not married	15 (17.2)	
Married	48 (55.2)	
Partner, not married	24 (27.6)	

During summer, older siblings (mean age = 11.2 years) reported, on average, 1069 active minutes and 1244 sitting minutes per week; younger siblings (mean age = 8.3 years) reported, on average, 1201 active minutes and 1368 sitting minutes per week. During fall, older siblings reported 927 mean active minutes and 1171 mean sitting minutes per week; younger siblings reported 909 mean active minutes and 1171 mean sitting minutes per week. Active minutes and MET-minutes did not differ between summer and fall time periods for older brothers, older sisters, or younger sisters, but younger brothers reported significantly fewer active minutes (mean = − 459.6; *p* = 0.01) and significantly fewer MVPA MET-minutes (mean = − 2261.7; *p* = 0.02) of physical activity per week during the fall than during summer (Table 3).

**Table 3:** Self-reported weekly physical activity and sedentary behavior by time period, sibling order, and gender

	Older sibling		Younger sibling	
	Brother (*n* = 42)	Sister (*n* = 45)	Brother (*n* = 44)	Sister (*n* = 43)
Active minutes, mean (std)				
Summer	1256.1 (724.0)	895.0 (506.7)	1546.4 (1055.6)	848.0 (697.8)
Fall	1086.6 (527.9)	777.4 (568.9)	1086.9 (749.7)	726.9 (541.5)
Δ	−169.5 (847.6)	−117.6 (835.5)	−459.6 (1075.2)	− 121.1 (827.9)
*P for change*	0.20	0.35	0.01	0.34
MVPA MET-minutes, mean (std)				
Summer	6570.1 (3914.0)	4405.1 (3213.3)	8092.5 (5847.2)	4402.8 (4018.3)
Fall	6217.7 (3073.2)	3864.1 (3114.8)	5830.8 (3897.3)	3795.1 (2956.4)
Δ	− 352.4 (4727.5)	−541.0 (4558.9)	−2261.7 (5981.0)	− 607.8 (4628.1)
*P for change*	0.63	0.43	0.02	0.39
Sitting minutes, mean (std)				
Summer	1325.0 (1043.1)	1168.8 (701.9)	1450.0 (1535.8)	1285.2 (1577.7)
Fall	1368.6 (1043.3)	987.9 (585.7)	1138.9 (682.6)	1203.5 (732.3)
Δ	43.6 (1090.2)	− 180.9 (938.8)	−311.1 (1756.0)	−81.7 (1682.4)
*P for change*	0.80	0.20	0.25	0.75
Screen time minutes, mean (std)				
Summer	1040.0 (859.6)	893.2 (652.0)	1048.0 (779.7)	828.4 (676.1)
Fall	959.3 (871.0)	543.9 (337.3)	641.0 (419.1)	759.0 (598.2)
Δ	−80.7 (919.2)	− 358.3 (752.6)	− 407.0 (891.8)	−69.4 (791.3)
*P for change*	0.57	< 0.001	0.004	0.57

Notes: Active minutes were defined as a 7-day total of moderate and vigorous physical activities (MVPA). The Compendium of Metabolic Equivalents (MET) for children was used to calculate MVPA MET-minutes, with moderate physical activity defined as 3–6 metabolic equivalents and vigorous physical activity defined as > 6.0 metabolic equivalents. Sitting minutes were defined as a 7-day total of sedentary activities such as homework or television viewing, and screen time minutes were a subset of sitting minutes that involved the use of media like television viewing or texting

**Fig. 1:**
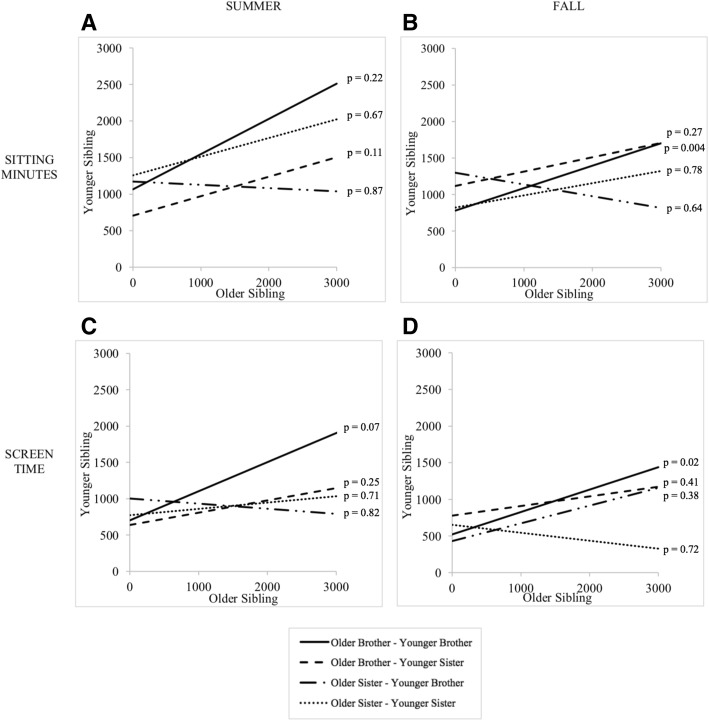
Comparison of older and younger sibling sedentary behaviors during summer (non-school) and fall (school). **a**. Sitting minutes per week during summer. **b**. Sitting minutes per week during fall. **c**. Screen time minutes per week during summer. **d**. Screen time minutes per week during fall

**Fig. 2:**
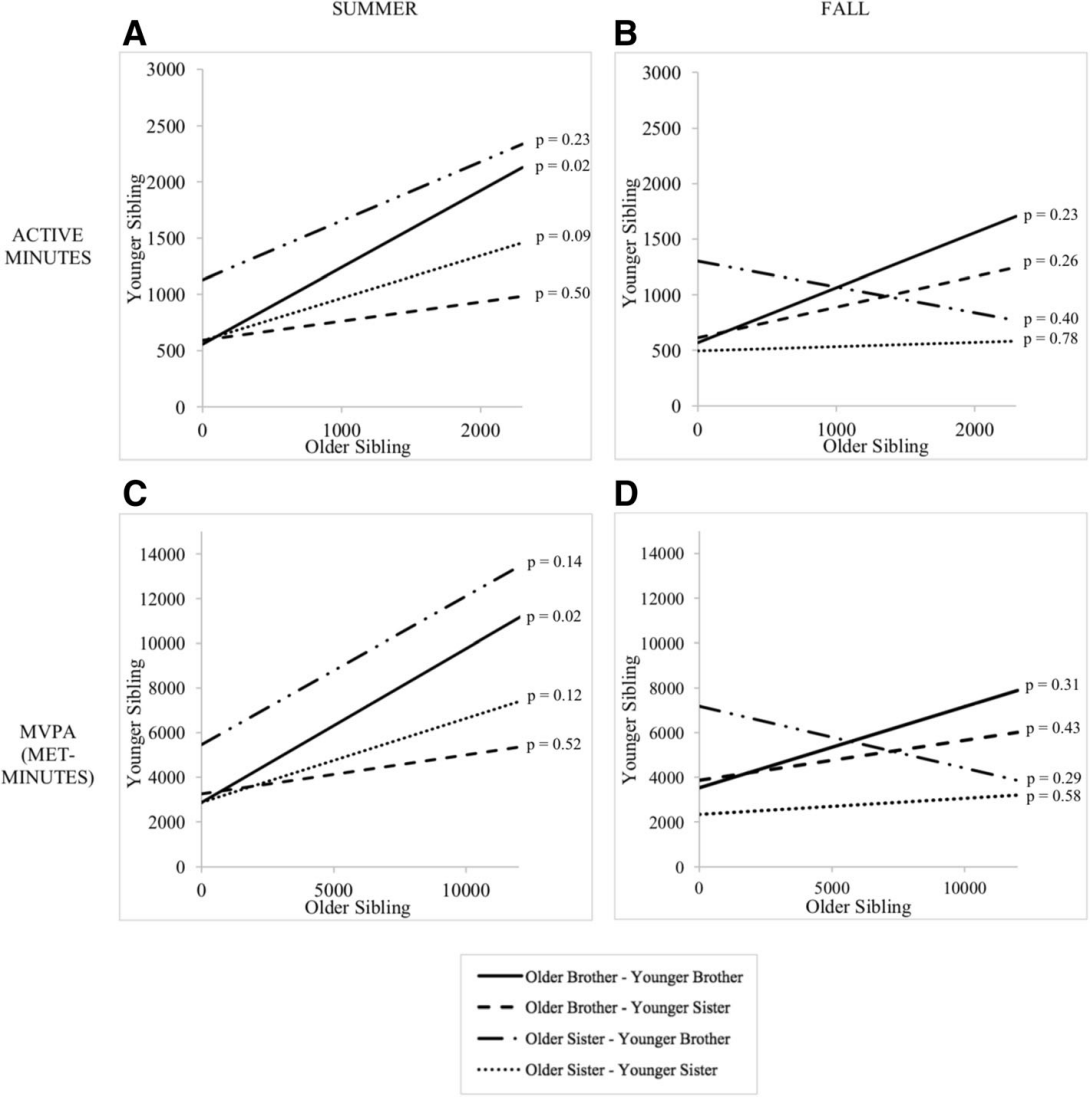
Comparison of older and younger sibling physical activity behaviors during summer (non-school) and fall (school). **a**. Active minutes per week during summer. **b**. Active minutes per week during fall. **c**. Moderate-to-vigorous physical activity (MVPA) metabolic equivalent (MET) minutes per week during summer. **d**. MVPA MET-minutes per week during fall

Within all 87 dyads, older sibling active minutes and MET-minutes were significant predictors of younger sibling active minutes (β = 0.44; *p* = 0.004) and MET-minutes (β = 0.45; *p* = 0.003), respectively, during summer but not fall; older sibling sitting and screen time minutes were significantly associated with younger sibling sitting (β = 0.23; *p* = 0.01) and screen time minutes (β = 0.23; p = 0.004), respectively, during fall but not summer (Table 4). After stratifying by gender dyad groups, younger brother behavior was strongly associated with older brother behavior at both time points. During fall, older brother active minutes and MET-minutes were significantly associated with younger brother active minutes (β = 0.68; *p* = 0.02; Adjusted R^2^ = 0.22) and MET-minutes (β = 0.69; p = 0.02; Adjusted R^2^ = 0.22), respectively; during summer, older brother sedentary time spent sitting and screen time minutes were significantly associated with younger brother sitting minutes per week (β = 0.31; *p* = 0.004; Adjusted R^2^ = 0.32) and screen time minutes per week (β = 0.31; p = 0.02; Adjusted R^2^ = 0.21), respectively. As shown in Figs. 1 and 2, there were no statistically significant relationships at the α = 0.05 level between older and younger behaviors for older brother-younger sister, older sister-younger brother, or older sister-younger sister groups.

**Table 4:** Within-dyad relationships between older sibling and younger sibling physical activity and sedentary behaviors by time period

		Younger sibling							
		Model: Active minutes		Model: MET-minutes		Model: Sitting minutes		Model: Screen time	
		B (SE)	p	B (SE)	p	B (SE)	p	B (SE)	p
Summer	Intercept	735.0 (183.5)	< 0.001	3801.6 (973.4)	< 0.001	913.0 (283.9)	0.002	754.1 (126.0)	< 0.001
	Older sibling								
	Active minutes	0.44 (0.14)	0.004						
	MET-minutes			0.45 (0.15)	0.003				
	Sitting minutes					0.37 (0.19)	0.05		
	Screen time minutes							0.19 (0.10)	0.07
	*Adjusted R* ^*2*^	*0.09*		0.09		0.03		0.03	
Fall	Intercept	779.1 (139.1)	< 0.001	4192.3 (702.1)	< 0.001	896.6 (124.0)	< 0.001	529.3 (78.4)	< 0.001
	Older sibling								
	Active minutes	0.14 (0.13)	0.28						
	MET-minutes			0.13 (0.12)	0.28				
	Sitting minutes					0.23 (0.09)	0.01		
	Screen time minutes							0.23 (0.08)	0.004
	*Adjusted R* ^*2*^	0.002		0.002		0.07		0.08	

Notes: Active minutes were defined as a 7-day total of moderate and vigorous physical activities (MVPA). The Compendium of Metabolic Equivalents (MET) for children was used to calculate MVPA MET-minutes, with moderate physical activity defined as 3–6 metabolic equivalents and vigorous physical activity defined as > 6.0 metabolic equivalents. Sitting minutes were defined as a 7-day total of sedentary activities such as homework or television viewing, and screen time minutes were a subset of sitting minutes that involved the use of media like television viewing or texting

## Discussion

The purpose of this study was to compare physical activity and sedentary behavior between older and younger siblings within Mexican-heritage sibling dyads across school year and non-school year time points and determine if the gender composition of sibling dyads modified the relationship between older and younger sibling behaviors. During the summer, older siblings reported 1069 mean minutes per week (or approximately 2.5 h per day) of physical activity and 1244 mean minutes per week (or approximately 3.0 h per day) of sitting, while younger siblings reported both more physical activity (1201 mean minutes per week or approximately 2.9 h per day) and more sitting time (1368 mean minutes per week or approximately 3.3 h per day). During fall, slightly fewer minutes of physical activity and sedentary behavior were reported for both older and younger siblings.

The Physical Activity Guidelines for Americans recommend children ages 6 to 17 participate in 60 or more minutes per day in aerobic activity, defined as either moderate- or vigorous-intensity activities such as unstructured active play and school recess activities like running and jumping [20]. Our findings with self-reported data on physical activity minutes per week indicate 82 and 79% (results not shown) of study participants in summer and fall, respectively, were active for at least 420 min per week, which is consistent with national guidelines for 60 min of activity per day. In contrast, national surveillance data indicate approximately 40% of youth and adolescents in the United States meet physical activity guidelines [21].

Older brothers, older sisters, and younger sisters reported more sitting time than physical activity time, and two-thirds of sitting time for all age-gender groups was spent using an electronic device such as television viewing, videogames, or texting. Results from our study appeared to indicate that youth participants reported more sitting time during the summer compared to the fall, although results for summer-to-fall change over time did not reach statistical significance at the α = 0.05 level. These findings are consistent with other work that has reported screen time is one of the largest groupings of sedentary behavior [22]. National survey data from 2015 indicate 8 to 12 year old children, on average, report 4 h of screen time per day [23]. Although the United States does not currently have national recommendations regarding sedentary behavior, other countries do have guidelines. The American Academy of Pediatrics recently established a policy statement on media use in children and adolescents that recommends limit setting and acknowledges the delicate daily balance between media use, physical activity, and sleep time [24]. Canada’s 24-h movement guidelines for optimal health benefits for children and youth (5–17 years of age) state that a “healthy 24 hours includes…no more than 2 hours per day of recreational screen time and limited sitting for extended periods” ([25] ^pg.S319^). Although children from lower-income homes have less access to devices like smartphones or tablets, national data indicate children from lower-income homes spend more time using media than children from higher-income homes [23], necessitating targeted sedentary reduction interventions for low-income families.

Younger siblings may emulate the behaviors of their older siblings. Specifically, our findings suggest that younger siblings appear to emulate the physical activity behaviors of their older siblings during non-school summer months and the sedentary behaviors of older siblings during school-time fall months. Few studies have evaluated sibling dyads and physical activity behaviors, and there is disagreement in the current literature about how sibling relationships effect physical activity levels between the siblings. One study found that older siblings participate in more physical activity than younger siblings [9], while another stated that older siblings are less physically active than their younger counterparts [10]. However, there does appear to be some consensus about the effect of gender. Sibling dyads that include a male sibling are more physically active, especially among brother-brother pairs [10] or among pairs with an older brother [26]. In our study, after stratifying the dyad comparisons by gender composition, the relationship between older and younger sibling behavior was particularly robust for older brother-younger brother dyads. Over 20% of the variance in younger brother activity during the fall and in younger brother sedentary behavior during the summer was explained by the respective behavior of the older brother. Our variance estimates are consistent with other work on the influence of male siblings [27]. These findings also support the social cognitive theory, suggesting that younger brothers may emulate the behaviors of their older brothers.

Family roles and cultural values may influence physical activity among sibships [9, 10, 26]. Mexican-heritage families report high levels of familism, or normative beliefs that the family is an important source of authority, support, guidance, and obligation [11]. Gender-typed traits like *machismo* (toughness, honor, responsibility) for boys and *marianismo* (collectivism, nurturance, passiveness) for girls is common in traditional Mexican culture [28, 29] and inversely related to other high-risk behavior like truancy [30]. Traditional gender-typed values for boys may provide more freedom outside the home and thus more opportunities for physically active outdoor play. In our study, at both time points, female children reported fewer active minutes and more sitting minutes than male children, which is consistent with gender-typed traits from Mexican-heritage family values. Our findings that female children are less physically active and more sedentary are also consistent with national surveillance on physical activity [21], suggesting culturally-appropriate physical activity interventions to increase physical activity and decrease sedentary time could be particularly useful for female children.

Other work has also highlighted larger social peer networks, such as friendships and peer groups. One study on adolescents’ sex-typed friendship experiences suggests older female siblings and her friends model interpersonal skills and emotional intimacy for younger siblings, while older male siblings and his friends model masculine leisure interests for younger siblings [31]. This work by Updegraff et al. [31] is notable because sibling dyads and a close friend of each sibling were evaluated with respect to intimacy, control, and personal qualities like interest in traditionally masculine and feminine leisure activities. Girls tended to rate relationships higher in intimacy, while boys reported more controlling behavior [31]. Findings suggested sex-typed friendship experiences were dependent upon the sex constellation of the sibling dyad. Younger sisters with older brothers appeared to value intimacy and younger brothers with older sisters appeared to value masculine interests [31] – both results supporting the de-identification theory. However, having a brother was also linked to a sister’s use of control strategies [31], supporting the social cognitive theory. Within the context of the present study, our findings indicate younger brothers emulate the physical activity patterns of their older brothers during summer months and the sedentary behaviors of their older brothers during the school year, supporting a social cognitive theory of sibling interaction. We did not observe a relationship between older sister-younger brother dyads or older brother-younger sister dyads, which is in keeping with previous work supporting the de-identification theory of interaction between gender discordant sibling pairs. Notably, we also did not observe a relationship – either positive or negative – between older sister and younger sister behaviors. These findings were surprising considering our findings for brothers, which seemed to support the social cognitive theory for gender concordant pairings. More work is needed to understand the role of sisters in Mexican-heritage families.

This study has several limitations. The gender composition of the dyads – older brother-younger brother, older brother-younger sister, older sister-younger brother, and older sister-younger sister – was evenly distributed and allowed us to examine birth order and gender while controlling age differences between older and younger siblings (due to eligibility criteria); however, a relatively small sample size of 174 children, or 87 sibling dyads, limited our ability to control for potential confounding variables in our regression models, which may have influenced our findings. For example, we did not examine the home environment, positions of the sibling dyads within a potentially larger sibship, or sibling intimacy. Although eligible families were similar in terms of geographic residence and ethnicity, the presence or absence of other family members, including additional older or younger siblings, may differ within our study population. Furthermore, other work has shown the older brother-younger sister dyad is the least intimate of sibling gender groups [32]. Future work may wish to consider sibling intimacy, conflict, or quality, in addition to the home environment and values such as familism, as factors that can influence the effect of older sibling behavior on younger sibling behavior [26]. We used a validated 7-day recall instrument with pictures to measure physical activity among child participants [16], but self-reported data is subject to recall bias and may be over-reported. Although we used winsorizing to correct for potential outliers and over-reporting, this method may mask the distribution of true behavior, which is not known without device-based (e.g., accelerometer) physical activity assessments.

Nevertheless, our study has notable strengths, including a within-family design and surveys during both an in-school and out-of-school time point. Siblings are the most frequent out-of-school companions for children and adolescents [33], suggesting the timing of school year may affect the strength of sibling influence on behaviors. In our study, as previously noted, younger siblings appear to emulate the physical activity behaviors of their older siblings during non-school summer months and the sedentary behaviors of older siblings during school-time fall months. More work is needed to replicate these findings longitudinally with a larger sample size. In addition, our ethnically-homogenous sample allowed us to identify unique patterns in behavior that can be used for culturally-appropriate intervention development among Mexican-heritage families. Hispanic populations, the majority of whom are Mexican, are the largest and fastest growing race/ethnic minority group in the United States [34], so understanding the health behavior patterns among families of this underserved population is an important direction for obesity- and chronic disease-related epidemiology and intervention research. These results may have broader applications for other low-income families as well, because differential access to health-promoting resources may be mitigated by strong family and sibling bonds [35].

## Conclusions

Our findings about sibling physical activity and sedentary behavior contribute to a surprisingly small body of literature on sibling health behaviors. Older brother-younger brother sibling dyad behaviors observed in our study support the importance of modeling, as posited in the social cognitive theory, and also confirm that like other U.S. children, these boys are more active than girls. However, more work is needed to better understand the complex relationships among sibships, gender, birth order, and social influences relating to health behaviors like physical activity and sedentary behavior among Mexican-heritage children. Interventions promoting Mexican-heritage youth physical activity should consider incorporating intervention components that build on cultural strengths like familism, particularly for male youth; provide opportunities for family and sibling engagement; and work to increase opportunities, skill building, meaningful modeling, and self-efficacy for girls.

## Abbreviations

MET: Metabolic equivalent
MVPA: Moderate-to-vigorous physical activity
